# Dinitrogen Fixation Is Restricted to the Terminal Heterocysts in the Invasive Cyanobacterium *Cylindrospermopsis raciborskii* CS-505

**DOI:** 10.1371/journal.pone.0051682

**Published:** 2013-02-06

**Authors:** Álvaro M. Plominsky, John Larsson, Birgitta Bergman, Nathalie Delherbe, Igor Osses, Mónica Vásquez

**Affiliations:** 1 Department of Molecular Genetics and Microbiology, Pontificia Universidad Católica de Chile, Santiago, Chile; 2 Department of Botany, Stockholm University, Stockholm, Sweden; Auburn University, United States of America

## Abstract

The toxin producing nitrogen-fixing heterocystous freshwater cyanobacterium *Cylindrospermopsis raciborskii* recently radiated from its endemic tropical environment into sub-tropical and temperate regions, a radiation likely to be favored by its ability to fix dinitrogen (diazotrophy). Although most heterocystous cyanobacteria differentiate regularly spaced intercalary heterocysts along their trichomes when combined nitrogen sources are depleted, *C. raciborskii* differentiates only two terminal heterocysts (one at each trichome end) that can reach >100 vegetative cells each. Here we investigated whether these terminal heterocysts are the exclusive sites for dinitrogen fixation in *C. raciborskii*. The highest nitrogenase activity and NifH biosynthesis (western-blot) were restricted to the light phase of a 12/12 light/dark cycle. Separation of heterocysts and vegetative cells (sonication and two-phase aqueous polymer partitioning) demonstrated that the terminal heterocysts are the sole sites for *nifH* expression (RT-PCR) and NifH biosynthesis. The latter finding was verified by the exclusive localization of nitrogenase in the terminal heterocysts of intact trichomes (immunogold-transmission electron microscopy and *in situ* immunofluorescence-light microscopy). These results suggest that the terminal heterocysts provide the combined nitrogen required by the often long trichomes (>100 vegetative cells). Our data also suggests that the terminal-heterocyst phenotype in *C. raciborskii* may be explained by the lack of a *patL* ortholog. These data help identify mechanisms by which *C. raciborskii* and other terminal heterocyst-forming cyanobacteria successfully inhabit environments depleted in combined nitrogen.

## Introduction

Cyanobacteria are photosynthetic organisms of great ecological importance due to their carbon fixation (photosynthesis) which is combined in several genera with N_2_ fixation (diazotrophy). Because the enzyme responsible for N_2_ fixation (nitrogenase) is irreversibly inactivated by O_2_, most diazotrophic filamentous cyanobacteria can only fix N_2_ under micro-oxic conditions (*e.g*. by fixing N_2_ in the dark or reducing their photosynthetic rates when fixing N_2_) [1]. Some filamentous cyanobacteria have solved this dilemma by differentiating a proportion of their vegetative cells into heterocysts, which are non-photosynthetic N_2_-fixing cells [2]. When combined nitrogen becomes depleted most heterocystous cyanobacteria differentiate 5–10% of their vegetative cells into heterocysts, which are individually spread at regular intervals along the trichomes (intercalary heterocysts) [3]. This regularity means that each heterocyst provides the nitrogen required by the ∼8–12 neighboring, non-N_2_-fixing vegetative cells [4]. Numerous studies detail the differentiation process of intercalary heterocysts [2] and the N_2_ fixation strategies operative in these cyanobacteria [4], [5], [6]. These studies have primarily focused on the model organism *Anabaena* sp. strain PCC 7120 (hereafter *Anabaena* PCC7120) and *Anabaena variabilis* strain ATCC 29413 (hereafter *A. variabilis* ATCC29413). However, some cyanobacteria differentiate heterocysts exclusively at the end of their trichomes (terminal heterocysts), such as cyanobacteria of the genera *Cylindrospermum* and *Cylindrospermopsis*. Few studies have addressed the physiology or mechanisms related to this terminal heterocyst phenotype [7], [8], [9]. Although their trichomes may be composed of more than 100 cells, these cyanobacteria differentiate at the most two heterocysts [7]. It has been shown that Δ*patA* (*all0521*) mutant of *Anabaena* PCC7120 only differentiates terminal heterocysts [10]. This suggests that *patA* is crucial for the differentiation of intercalary heterocysts and that the differentiation of terminal heterocysts is regulated differently. However, this mutant grows poorly when lacking combined nitrogen, suggesting that the terminal heterocysts, in spite of being able to fix nitrogen, cannot satisfy the levels of nitrogen demanded by the whole trichome [11].

**Figure 1:**
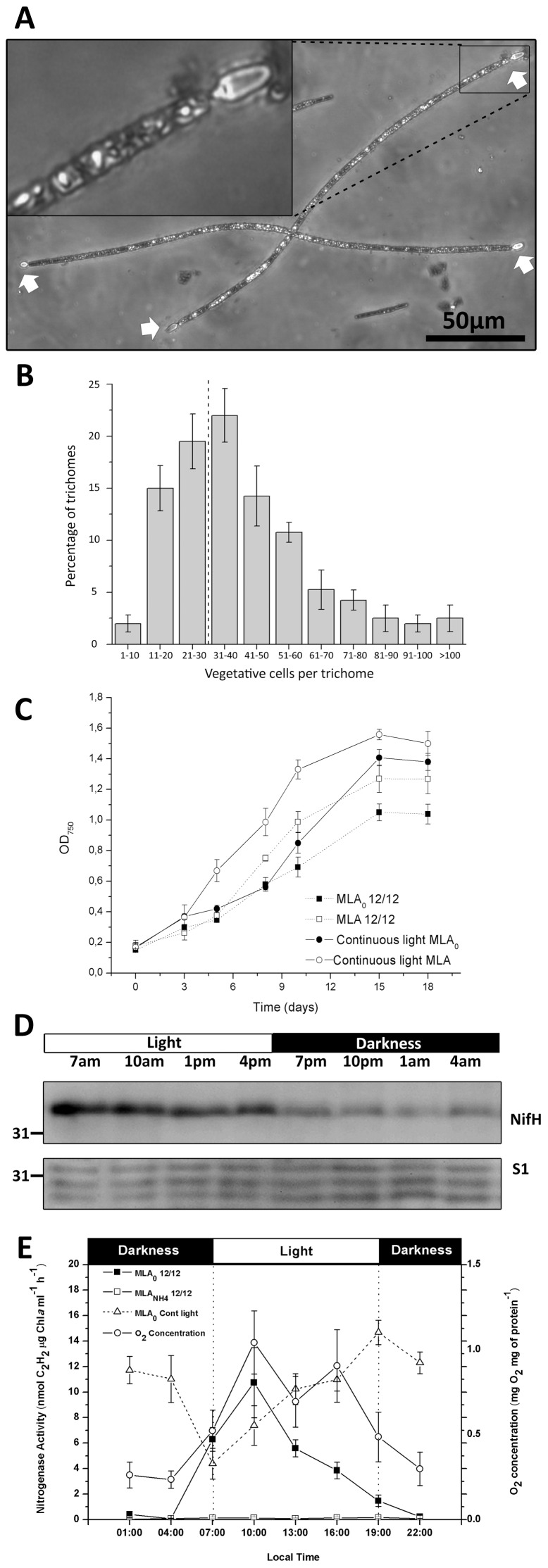
The cyanobacterium *C. raciborskii* grown under diazotrophic conditions. (A) Light micrograph of *C. raciborskii* trichomes, magnified 40x. Note the terminal heterocysts at the end of the trichomes (white arrows); one heterocyst is magnified. (B) Percentage of trichomes with 10 to >100 vegetative cells are grouped according to their trichome length (no. of vegetative cells per trichome). The bars give the average numbers in 400 trichomes from 4 independent cultures. Standard deviation is given. The dashed line denotes the point where all trichomes have developed two end heterocysts. (C) The growth of *C. raciborskii* cultures, grown with 2 mM of NO_3_^−^ to an OD_750_ of 0.2, washed and transferred to new medium under the denoted light regime. Each point corresponds to the mean of 4 biological replicates. Standard deviation is given. (D) Western-blot analysis of NifH expression during a 12L/12D cycle (above). The NifH western-blot membrane was stripped and re-labelled with an antibody against the 30S ribosomal protein S1, revealing protein levels loaded in each well (below). The closest molecular marker band is indicated, and black and white bars represent the light and dark phase of the cycle. (E) Nitrogenase activity was analyzed by ARA for *C. raciborskii* under diazotrophic growth and continuous light (MLA_0_ Cont light), diazotrophic growth and 12L/12D cycle (MLA_0_ 12/12), and grown in MLA_N_ under 12L/12D cycle (MLA_N_ 12/12). Oxygen concentration was measured for MLA_0_ 12/12 cultures and normalized to protein content. Each point corresponds to the mean of three biological replicates where standard deviation is shown.

In contrast to *Anabaena* PCC7120, which has only one type of nitrogenase, the intercalary heterocystous cyanobacterium *A. variabilis* ATCC29413 expresses one type of nitrogenase in the heterocysts under both aerobic and anaerobic conditions (encoded by the *nif1* gene cluster), while a second nitrogenase is expressed in the vegetative cells under anaerobic conditions only (encoded by the *nif2* gene cluster) [6]. Likewise, numerous filamentous non-heterocystous cyanobacteria are able to express the *nif* genes in the vegetative cells (*e.g*. under darkness; [1]). Hence, we hypothesized that the terminal heterocysts of *Cylindrospermopsis* may rely on both a *nif1*-like nitrogenase (in the heterocysts) and the co-support of a *nif2*-like nitrogenase expressed during the dark phase of the cycle (∼low oxygen) in their vegetative cells. However, the genome sequence of *Cylindrospermopsis raciborskii* CS-505 (hereafter *C. raciborskii*), the first sequenced cyanobacterium with terminal heterocysts (Fig. 1A), revealed the presence of a single *nif1*-like gene cluster [12]. Thus, the presence of an additional *nif*–cluster was excluded, which suggests that other mechanisms must be operative. Interestingly, *C. raciborskii* shows a highly successful ecological competence and has recently been observed to radiate from its endemic subtropical environment into limnic water bodies in temperate latitudes worldwide [13], [14]. The ecological success of this cyanobacterium may be attributed not only to its facultative diazotrophic capacity [15], but also to its capacity to differentiate spore like cells (akinetes) under adverse conditions [16], [17] and the existence of multiple toxin producing phenotypes within the *Cylindrospermopsis* genus. Some strains produce the hepatotoxin cylindrospermopsin [16], and others produce the neurotoxin saxitoxin [17], while some strains lack toxin production entirely. The toxin production, besides representing threats to human health and livestock, may render additional competitive advantages in nature [18].

Here we have analyzed the strategy employed by *C. raciborskii* to survive combined nitrogen depletion while differentiating only terminal heterocysts. Our analysis focused on determining nitrogenase expression in time and space along the trichomes of *C. raciborskii*, and discovering the genomic basis determining the terminal heterocyst phenotype (*i.e*. preventing the formation of intercalary heterocysts in this cyanobacterium).

## Results

### Diazotrophic Growth, *nif*H Levels and Nitrogenase Activity

Microscopic examinations verified that *C. raciborskii* trichomes differentiate exclusively terminal heterocysts when grown lacking combined nitrogen (Fig. 1A). In diazotrophic cultures of *C. raciborskii* (OD_750_ of 0.55–0.65), terminal heterocysts may be separated by a few or even up to >100 vegetative cells (Fig. 1B). One heterocyst always starts to differentiate at one end of the trichome and when the trichome has elongated to approx. 30 cells, another heterocyst starts to differentiate at the opposite end (Fig. 1B). In these diazotrophic cultures (OD_750_ of 0.55–0.65), 81% (SD ± 2.97) of the trichomes developed two end-heterocysts, and each heterocyst contained an average of 22.7 (SD ±3.91) vegetative cells. However, up to 40.1% (SD ± 10.07) of the *C. raciborskii* trichomes were composed of considerably more than 50 vegetative cells each (Fig. 1B). Notably, while the terminal heterocyst phenotype persisted, the number of vegetative cells per trichome increased as the cultures approached the stationary phase (data not shown). This is in stark contrast to the situation in *Anabaena* PCC7120, in which there are generally 7–15 vegetative cells per heterocyst [19].

Several approaches were used to determine whether the terminal heterocysts of *C. raciborskii* are the sole sites for N_2_ fixation, and whether N_2_ fixation may take place in the vegetative cells under any conditions (*e.g*. in darkness). First, we compared diazotrophic growth under continuous light and 12/12 light/dark (12L/12D) cycles, respectively (Fig. 1C). The cultures were grown in 2 mM NO_3_^−^ as an N-source (MLA medium) under continuous light or under 12L/12D cycles until they reached an OD_750_ of ∼0.2. Then they were washed and half of the cultures were maintained in the MLA medium, while the other half were resuspended in medium lacking combined nitrogen sources (MLA_0_). All cultures were incubated under their corresponding light regime until they reached the stationary phase (Fig. 1C). The growth of *C. raciborskii* was stimulated under all conditions, particularly under continuous light, both in the presence and absence of a combined nitrogen source. However, diazotrophic growth was considerably lower under the 12L/12D regime, clearly indicating that darkness neither supports nor enhances diazotrophic growth in *C. raciborskii*.

To determine when nitrogenase is synthesized in diazotrophic cultures grown under 12L/12D cycles, protein levels were determined by western-blots based on an antibody raised against NifH (Fe-protein; approx. 34 kDa). As seen in Figure 1D (upper image), higher NifH levels were synthesized in the light compared to the dark phase of the cycle. Similar NifH levels were observed in three separate diel studies (data not shown). The same western blot membranes were then stripped and re-labelled with an antibody raised against the 30S riboprotein S1. As seen in Figure 1D (lower image), this loading control shows that similar protein levels were present at each time point, supporting the NifH protein variations observed.

Next, the nitrogenase activity profile of *C. raciborskii* was analyzed by the acetylene reduction assay (ARA; Fig. 1E) under three growth conditions: i) diazotrophic growth under continuous light (MLA_0_ cont. light); ii) diazotrophic growth under a 12L/12D cycle (MLA_0_ 12/12); and for comparison, iii) non-diazotrophic growth conditions under 12L/12D cycle (MLA_N_ 12/12). Additionally, the oxygen levels of the MLA_0_ 12/12 cultures were monitored in parallel. As expected, no nitrogenase activity was observed in the non-diazotrophic cultures. In the MLA_0_ 12/12 cultures the nitrogenase activity showed a distinct light dependent pattern, being close to zero in the dark phase while it peaked 3 h into the light phase and then decreased when approaching the new dark phase of the cycle. Notably, the oxygen concentration of the cultures also increased and remained high during the light period, which may explain the decrease in nitrogenase activity in the MLA_0_ 12/12 cultures beyond the peak at 3 hours (Fig. 1E). Under continuous light, the nitrogenase activity fluctuated but was pronounced at all time points analyzed (Fig. 1E), stressing the light dependence of the nitrogenase activity. These results demonstrate that the nitrogenase activity of *C. raciborskii*, as in other examined heterocystous cyanobacteria, is dependent on energy generated during the light phase of the cycle.

### NifH Levels and *nif*H Expression in Heterocysts and Vegetative Cells

**Figure 2:**
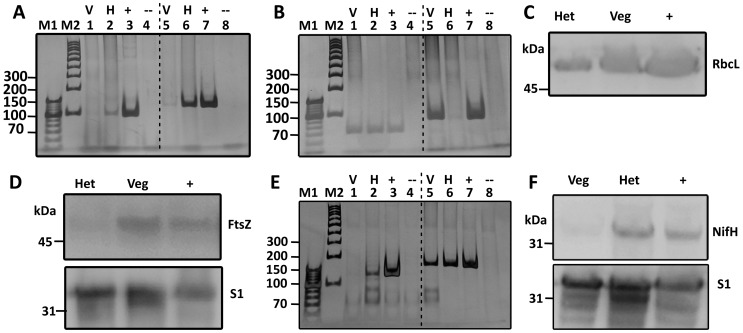
Cell enrichment purity assessment and nitrogenase expression in heterocyst and vegetative cells of *C. raciborskii*. Heterocysts and vegetative cells of *C. raciborskii* were separated, proteins were extracted and cDNA was generated for both cell fractions. (A) RT-PCR amplification for *dev*A (lanes 1–4) and *hgl*D (lanes 5–8). (B) PCR amplification of *rbc*S (lanes 1–4) and *fts*Z (lanes 5–8). (C) RbcL levels. (D) FtsZ (upper) and S1 protein levels (lower). (E) RT-PCR amplification of *nif*H (lanes 1–4) and a fragment of the 16S rRNA (lanes 5–8). (F) NifH levels (upper) and S1 protein levels (lower). Western-blot membranes were stripped and re-labelled with the 30S ribosomal protein S1 to verify loading similarities. Protein levels and selected gene expression levels were determined in enriched heterocysts (Het/H) or vegetative cell (Veg/V) fractions. Protein extracts from non-fractionated trichomes of *C. raciborskii* were used as a positive control (+). The closest molecular marker band is indicated. *C. raciborskii* DNA, and the RNA used to generate the cDNA, were used as positive (+), and negative (−) controls respectively. M1 and M2 denote DNA size markers (bp).

To examine to what extent the vegetative cells are able to express and synthesize nitrogenase, vegetative cells and heterocysts of a *C. raciborskii* culture grown under continuous light were analyzed separately. For this purpose, a two-phase aqueous polymer partition of the two cell types was performed. When analyzed by light microscopy (LM), the two cell fractions showed an enrichment of ∼95% per cell type (Table 1). The two cell fractions were then subjected to both RT-PCR and western-blot analyses. In order to validate the results from the two cell fractions, both heterocyst and vegetative cell markers were included. The transcripts of *devA* and *hglD* [20], [21] were used as markers for heterocysts. As seen in Figure 2A, these transcripts were detected only in the heterocyst enriched fractions. The transcripts of *rbcS*, encoding the small subunit of the RuBisCO, were used as a marker for vegetative cells [5]. Unexpectedly, both cell fractions showed presence of *rbcS* transcripts (Fig. 2B). This was verified by western-blots on whole cell protein extracts using an antibody against RbcL, encoding the large subunit of the RuBisCO (Fig. 2C). Further examination of the heterocyst fractions by epifluorescence-LM revealed the presence of immature heterocysts as verified by their retention of high levels of chlorophyll *a* (Chl*a*) and phycoerythrine autofluorescence (data not shown). Thus, the RuBisCO detected in the heterocyst enriched fractions (Fig. 2B and 2C) was most likely due to the presence of immature heterocysts and not to vegetative cell contaminations. To confirm this hypothesis, transcripts of the cell division-related *ftsZ* gene (filamenting temperature-sensitive mutant Z) and expression levels of the FtsZ protein were also examined. FtsZ levels are typically low or non-detectable in terminally differentiated non-dividing heterocysts but high in dividing vegetative cells [22], where the protein (molecular mass of 50 kDa) assembles into a ring at the site of the growing new septum. However, using this marker, it became clear that both *ftsZ* transcripts and the FtsZ protein were only detected in the vegetative cell fractions (Fig. 2B and 2D), which further shows the dominance of heterocysts in the heterocyst enriched fraction.

**Table 1:** Heterocyst and vegetative cell enrichment assessment.

Phase of the partitioning system	Cell type	Cell count*
Upper	Heterocysts	4.3±2.2
	Vegetative cells	95.6±2.2
Lower	Heterocysts	97.2±0.6
	Vegetative cells	2.7±0.6

Heterocyst and vegetative cells were physically separated, and the cells counted (in LM) for each separate cell enrichment.

* Values correspond to the mean of 100 cells of each phase ± SD of 4 independent cell partitions (n = 400).

Next, the expression of *nifH* and the levels of NifH in both cell fractions were examined. The expression of *nifH* was detected exclusively in the heterocysts of *C. raciborskii* (Fig. 2E), and western-blots revealed high levels of NifH in the heterocyst fractions compared to the vegetative cell fractions, which lacked this protein (Fig. 2F; upper image). These results were reproduced in three independent experiments (data not shown). When the same membranes were stripped and re-labelled for loading control assessment with the 30S riboprotein S1 antibody (Fig. 2F; lower image), it was evident that protein levels from the heterocyst and vegetative cell fractions were similar.

### Immunolocalization of Nitrogenase During a 12L/12D Cycle

**Figure 3:**
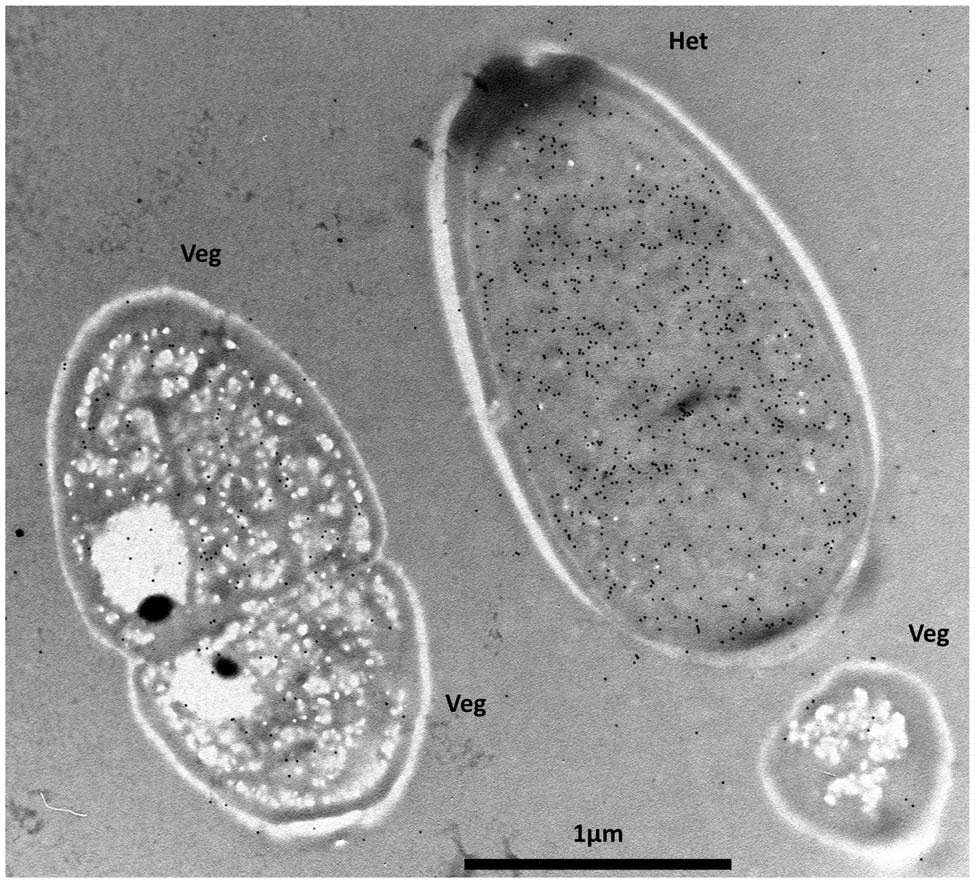
Immunogold-TEM localization of NifH in sectioned cells. Cultures grown in MLA_0_ were harvested 3 hours into the light phase in 12L/12D cycle. Representative micrographs of a heterocyst (Het) and vegetative cells (Veg) are shown after being subjected to an anti-NifH antibody and detected by a secondary antibody conjugated to10 nm gold particles. The corresponding label intensity for each cell type is given in Table 2.

**Figure 4:**
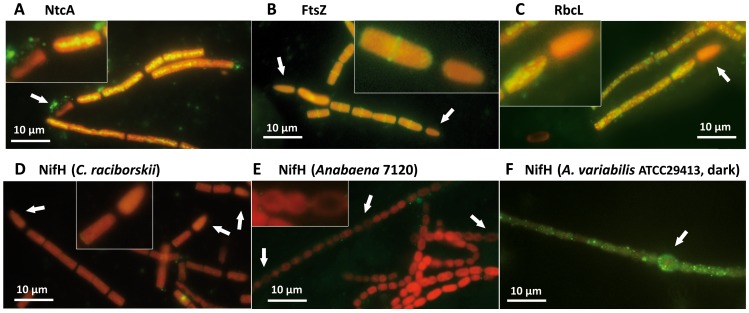
Immunofluorescence-LM of proteins in intact trichomes. Cultures grown in MLA_0_ and a 12L/12D cycle were sampled 3 h after the beginning of the light phase. Immunofluorescence was performed on *C. raciborskii* using antibodies against (A) NtcA, (B) FtsZ, (C) RbcL proteins and against the NifH protein in both (D) *C. raciborskii* and (E) *Anabaena* PCC7120. (F) *A. variabilis* ATCC29413 grown in BG11_0_ and a 12L/12D cycle was used as a positive control for NifH (*nif*2 nitrogenase) presence in vegetative cells (when sampled 4 h in the dark phase). Arrows indicate the positions of heterocysts.

These results suggest that *nifH* expression and NifH localization are restricted to terminal heterocysts for both cultures grown under a continuous light regime and those grown under a 12L/12D cycle. Because the cell isolation procedure takes longer to perform than the length of the nitrogenase activity peak (see Fig. 1B), intact sectioned trichomes were also tested using transmission electron microscopy coupled to gold conjugated antibodies (immunogold-TEM) targeting NifH. To determine nitrogenase localization during peak nitrogenase activity (Fig. 1E), trichomes were sampled 3 h into the light phase from cultures grown under 12L/12D cycles. Diazotrophic *Anabaena* PCC7120 was used to determine a comparative immuno-TEM-labelling baseline of NifH levels in heterocysts, as well as as biological background levels of the non-NifH vegetative cells. In both cyanobacteria the cross-sectioned heterocysts showed higher levels of NifH label compared to the vegetative cells (Table 2; Fig. 3). To determine whether these differences were statistically significant, NifH levels in heterocysts and vegetative cells of both cyanobacteria were subjected to two-way analysis of variance (ANOVA) testing between the same cell types, different cell types, and the two different species. The only statistically significant differences were between the NifH levels in heterocysts compared to the vegetative cells (F-value [1, 88]  = 167.99; p-value ≤0.001) (Table S1). Notably, the heterocysts of *Anabaena* PCC7120 and of *C. raciborskii* showed the same NifH levels (*i.e*. statistically not different; Table S1). Although some randomly distributed gold label was detected in the vegetative cells of *C. raciborskii*, this most likely represents biological background due to its low level in vegetative cells compared to heterocysts (Fig. 3). The low NifH levels detected by immunogold-TEM in the vegetative cells, suggested the potential existence of nitrogen-fixing diazocyte-like cells along the trichomes of *C. raciborskii* as in the cyanobacterium *Trichodesmium* [23], [24], [25]. Diazocyte cells are capable of carrying out N_2_ fixation in light, although they lack the protective thick cell walls characteristic of mature heterocysts [23], [24], [25]. Thus, we next assessed the presence of nitrogenase *in situ* along intact (non-sectioned) trichomes of *C. raciborskii* by *in situ* immunofluorescence-LM. *C. raciborskii* cultures grown under 12L/12D cycle were again sampled during peak nitrogenase activity, and key antibody markers were used to localize immunofluorescence labels inside permabilized heterocysts and vegetative cells. An antibody against the NtcA protein, a transcription factor sensing the nitrogen status of the vegetative cells in cyanobacteria [26], was used as a positive marker for heterocysts. Although NtcA is present in all the cells of the *C. raciborskii* trichome when induced by the depletion combined nitrogen, it is present at even higher levels in developing heterocysts [27]. The specificity of the antibodies raised against NtcA was verified by western-blot, using whole protein extracts from diazotrophic *C. raciborskii* cultures, revealing a single peptide of a molecular mass of 25 kDa (data not shown). Additionally, the antibodies against FtsZ and RbcL were used as positive markers for vegetative cells. Unexpectedly it was not possible to detect the NtcA fluorescence label inside heterocysts (Fig. 4A). However, immunofluorescence-LM analyses were positive for NtcA, FtsZ and RbcL in the vegetative cells of *C. raciborskii* (Fig. 4A, 4B and 4C). The NtcA protein showed a strong centroplasm localization signal, as expected, (Fig. 4A [27]). The FtsZ protein was also clearly organized into cell division rings in the actively dividing cells (Fig. 4B), and the RbcL immunofluorescence label was apparent in carboxysome-like subcellular structures spread over the cell interior (Fig. 4C). Finally, when NifH was assessed, no immunofluorescence-LM signal was detected in vegetative cells of *C. raciborskii* (Fig. 4D), strongly arguing against the expression of *nif* genes in this cell type. Likewise, NifH immunofluorescence labeling was not observed in the vegetative cells of *Anabaena* PCC7120 serving as a control (Fig. 4E). Interestingly, the NifH2 label [28] was apparent in vegetative cells of *A. variabilis* ATCC29413 when examined 4 hours into the dark phase of 12L/12D cycles (Fig. 4F). We therefore conclude that the low NifH levels detected by immunogold-TEM in vegetative cells of *C. raciborskii* corresponded to biological background label, as was also verified by the absence of NifH immunofluorescence label in the vegetative cells of *C. raciborskii*.

**Table 2:** Relative quantities of NifH in intact trichomes of *C. raciborskii* and *Anabaena* PCC7120 assayed by immunogold-TEM localization.

Organism	Cell type	Label intensity* (gold particles µm^−2^)
*C. raciborskii*	Heterocysts	194.9±88.3
	Vegetative cell	28.8±13.5
	Background	3.2±1.9
*Anabaena PCC7120*	Heterocysts	188.6±85.3
	Vegetative cells	18.7±13.2
	Background	4.0±3.0

*C. raciborskii* and *Anabaena* PCC7120 grown in MLA_0_ were sampled 3 h after the start of the light phase of the 12L/12D cycle, and immunogold-TEM localization of the NifH protein was performed.

* Values are means ± SD of a total of 23 cells (or different images for background label count) from 3 independent cultures. Background denotes the formvar covered areas on the TEM grids lacking cells (heterocysts and vegetative cells), the *i.e*. non-biological background.

### Genomic Background Determining Terminal Heterocyst Morphology

The genome of *C. raciborskii* lacks several genes related to heterocyst differentiation and heterocyst pattern regulation [12]. Still, it retains *patA, patL* and *hetF* homologs, which are known to be related to these functions [10], [11], [29], [30]. As a first approach to determine if the *C. raciborskii* homologs conserve their functions in heterocyst pattern regulation, we analyzed their phylogenetic relationship with the homologs characterized in *Anabaena* PCC7120.

**Figure 5:**
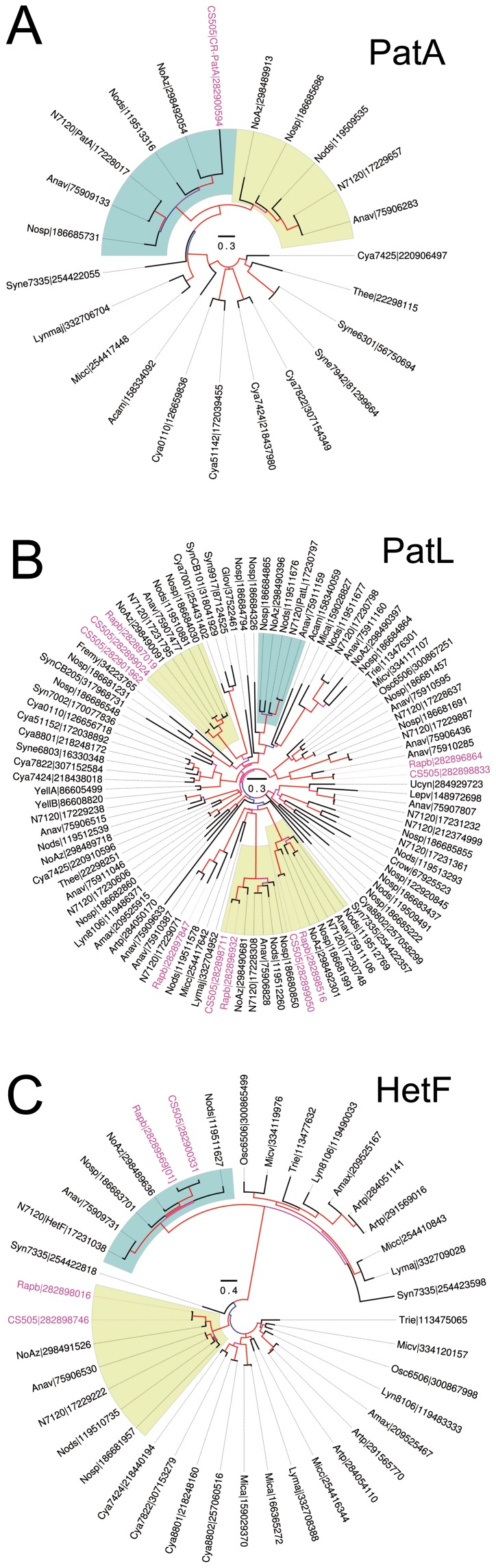
Phylogenetic analysis of PatAL and HetF homologs in *C. raciborskii*. Maximum-likelihood phylogenetic trees of (A) PatA, (B) PatL, and (C) HetF. Branches are colored by their support value (Shimodaira-Hasegawa test, low [blue] to high [red]). *C. raciborskii* and *R. brookii* sequences are colored in pink for clarity. Clades including the canonical heterocyst differentiation proteins are shaded in green while non-canonical heterocystous clades are shaded in yellow. Scale bar shows expected substitutions per site. In (C), the “[01]” suffix for Rapb|28289569 indicates that the sequence is a concatenation of two neighboring ORFs (see Information S1 for details).

The *Anabaena* PCC7120 PatA sequence (hereafter N7120-PatA, 379 aa; encoded by *all0521*), was used to retrieve a total of 23 homologous gene sequences from other cyanobacteria (Table S2). All homologs contained the conserved PatA N-terminal (PATAN) domain, and the CheY-like phosphoacceptor (receiver, REC) domain at the C-terminal end [31]. The resulting PatA phylogenetic tree showed that the *C. raciborskii* homolog (CR-PatA) is localized in the same clade as N7120-PatA and PatA sequences from intercalary heterocystous cyanobacteria (Fig. 5A, green shading). Identical topologies were obtained when analyzing the PATAN and REC domains separately (data not shown). Additionally, whole genome alignments show highly conserved synteny around the CR-PatA and the intercalary heterocystous PatA sequences (data not shown). Notably, CR-PatA (308 aa) has the shortest and least conserved sequence within its clade, sharing between 37–49% identical residues with PatA sequences from intercalary heterocystous cyanobacteria (which have 61–95% pairwise identity between themselves). Interestingly, a second PatA homolog was retrieved from all heterocystous cyanobacteria (Fig. 5A, yellow shading), except for *C. raciborskii*.

Analysis of synonymous and non-synonymous substitutions for the heterocystous *patA* sequences indicated that CR-*patA* is evolving under purifying selection and is thus likely to be functional. However, the shorter linker region between the PATAN and REC domains of CR-PatA, which is 20 aa compared to 55–91 aa in the PatA sequences of intercalary heterocystous cyanobacteria (Fig. S1), suggests that although the exact nature of the regulation of PatA in heterocystous cyanobacteria is unknown, this truncation in CR-PatA might limit proper interaction of the two domains in *C. raciborskii*.

*Anabaena* PCC7120 PatL (hereafter N7120-PatL, 496 aa), encoded by *all0521*, contains a region of several pentapeptide repeats at its C-terminus. A total of 1001 homologs in cyanobacteria were retrieved using N7120-PatL as query sequence. A subset of PatL homologs, including single best-scoring homologs from non-heterocystous cyanobacteria, all sequences from heterocystous cyanobacteria (Table S2), and all five paralogs in *C. raciborskii* (CR-PatL parologs), were used to construct a PatL phylogenetic tree (Fig. 5B). The resulting phylogenetic tree shows that none of the five CR-PatL parologs (Fig. 5B, pink letters) are placed in the same clade as N7120-PatL or its best-scoring homologs among heterocystous cyanobacteria (Fig. 5B, green shading). Additionally, there was no synteny around the CR-PatL paralogs and N7120-PatL in its best-scoring homologs among heterocystous cyanobacteria. These results suggest that the CR-PatL paralogs are most likely not involved in heterocyst pattern formation.

To analyze HetF homologs among cyanobacteria, sequences from *Anabaena* PCC7120, *Raphidiopsis brookii* D9 and *Trichodesmium erythraeum* passed a comprehensive manual editing and selection procedure (see Information S1). The alignment of the HetF-homologs showed that all sequences contained a conserved His-Cys dyad, which constitutes the active site residues of cysteine proteases (including caspases) [32]. Our phylogenetic analysis showed that one of the *R. brookii* and *C. raciborskii* HetF paralogs (RB-HetF and CR-HetF respectively) clustered together with the homologs of heterocystous cyanobacteria (Fig. 5C, green shading). Notably, all HetF homologs of this group, except for RB-HetF, contain a predicted transmembrane helix at approximately residue 550 (although with a lower probability in *Lyngbya* sp. PCC 8106; data not shown). No such transmembrane region was found in the homologs of the other major clades (Fig. 5C). These results suggest that CR-HetF might have a conserved role in heterocyst pattern formation and that the transmembrane region of HetF might be important for heterocyst differentiation.

## Discussion

Despite its restricted heterocyst frequency, *C. raciborskii* is an ecologically successful freshwater cyanobacterium which in recent decades has rapidly spread from tropical and sub-tropical to temperate aquatic regions [13], [14], [33], *e.g*. from Greece [34], via Hungary, Germany, Austria, France and recently to Portugal [13], [35], [36], [37], [38], [39]; as well as to Canada and New Zealand [40], [41]. Increasing water temperatures (‘climate change’) and ecotype selection have been proposed to explain this rapid change in the distribution of the toxin-producing *C. raciborskii*. The ecological competence of *C. raciborskii* has also been attributed to its facultative diazotrophy, which allows it to compete efficiently in environments with high fluctuations in combined nitrogen availability [15]. Despite its novelty and the ecological success of *C. raciborskii*, nitrogen fixation in terminal heterocystous cyanobacteria has until now largely remained unstudied. Our investigation shows that *C. raciborskii*, grown under combined nitrogen depletion and 12L/12D cycles, performs NifH biosynthesis and presents its highest nitrogenase activity in the light period (Fig. 1). Additionally, our data demonstrates that *C. raciborskii* restricts *nifH* expression and NifH biosynthesis to its two terminal heterocysts, irrespective of trichome length (Figs. 2, 3 and 4). Thus, the possibility that *C. raciborskii* compensates for lack of interspersed heterocysts by fixing N_2_ in the vegetative cells in the dark can be discarded, as verified by our localization of nitrogenase in intact trichomes (Fig. 4).

The question that follows is how *C. raciborskii* is able to sustain the long trichomes (often >100 vegetative cells long) with enough nitrogen with only two terminal heterocysts per trichome (Fig. 1A). When compared to *Anabaena* PCC7120, which has one heterocyst every 7–15 vegetative cells [19], both show similar NifH levels in their heterocysts (Table 2). Although *Anabaena* PCC7120 differentiates a higher frequency of heterocysts per trichome compared to *C. raciborskii*, both cyanobacteria showed similar maximum nitrogenase activity rates at their peak (12L/12D cycles): 8.37 (SD±1.98) and 10.20 (SD±1.43) nmol C_2_H_4_ per mg of protein mL^−1^ h^−1^ for *C. raciborskii* and *Anabaena* PCC7120, respectively (n = 3 cultures with three generations of diazotrophic establishment and an OD_750_ of 0.61 SD±0.05 and 0.63 SD±0.04 respectively). This finding points to a higher nitrogenase activity in the cyanobacterium with terminal heterocysts. Since our data clearly show that the sole sites for N_2_ fixation are the terminal heterocysts, *C. raciborskii* has to solve the problem of how to mobilize the fixed N_2_ from its terminal heterocysts to all other cells of its often long trichomes. One possibility is that *C. raciborskii* might possess a more efficient transport mechanism along its trichomes, perhaps combined with a more efficient detainment of the fixed N_2_ within the trichomes.

*C. raciborskii* has the smallest sequenced genome described for any sequenced heterocystous cyanobacterium (3.88 Mbp [12]). It is in fact approaching or even smaller than, the genome size of some unicellular diazotrophic cyanobacteria such as species within the genera *Cyanothece* and *Crocosphaera* [42]. Indeed, whole genome phylogeny suggests that *C. raciborskii* belongs to a clade of cyanobacteria characterized by shrinking genomes, which however seem to have originated from an ancestor with intercalary heterocysts and an estimated genome size of 5.65 Mbp [42], [43]. Interestingly, *R. brookii*, the closest relative to *C. raciborskii* with a sequenced genome, has an even smaller genome (3.18 Mbp [12]) and lacks heterocysts as well as N_2_ fixation activity altogether. In *Anabaena* PCC7120 a terminal heterocyst phenotype is apparent when deleting *patA* [10], [11] and *patL* [29]. Also, the terminal heterocyst phenotype of the *patA* mutant is reverted by ectopic expression of *hetF*, indicating that HetF exerts its function downstream of PatA [30]. Our phylogenetic analysis of the PatA, PatL and HetF homologs (Fig. 5) provides additional support for the close relatedness between *C. raciborskii*, *R. brookii* and the third member of this shrinking genome clade, a symbiont of the fern *Azolla*, ‘*Nostoc azollae*’ 0708 [12], [42], [43]. Also, in addition to the loss of several genes with functions in heterocyst differentiation and pattern regulation, the terminal heterocyst phenotype of *C. raciborskii* correlates with the lack of a *pat*L paralog, and not to the absence of *patA* or *hetF*. It has been suggested that genome reductions in *C. raciborskii* and *R. brookii* may also be a consequence of interactions with yet unidentified eukaryotic hosts. An alternative explanation is that this may simply represent another example of reductive genome evolution [12], [44], a strategy used by the globally wide-spread and ubiquitous marine unicellular cyanobacterial genera *Prochlorococcus* and *Synechococcus* [42], [45]. One of the few other examples of the terminal heterocyst phenotype of *C. raciborskii* are the so called primordia [46]. This is an intermediate life-stage between the small-celled motile hormogonia (functioning as either escape or plant colonization units), and fully mature vegetative trichomes of the heterocystous, often symbiotic, genus *Nostoc* [46]. However, both hormogonia and primordia are, as opposed to *C. raciborskii*, transient stages in the life span of *Nostoc* spp. In spite of the more severe genome streamlining in *R. brookii*, this closely related strain has more genes involved in “aa transport and metabolism” and “coenzyme transport and metabolism” than *C. raciborskii* [12]. The loss of heterocysts and N_2_ fixation in *R. brookii* has been suggested to be due to a consistent availability of dissolved inorganic or organic nitrogen sources in its natural environment [15]. Our analysis also revealed that, although RB-HetF is closely related to CR-HetF and all homologs from heterocystous cynanobacteria, it lacked the conserved transmembrane region present in all representatives of this clade. Additionally, the fact that RB-HetF is represented by two neighboring genes (Information S1) might reflect the ongoing streamlining of *R. brookii*.

Taken together, we here show an exclusive localization of the nitrogenase enzyme in the heterocysts in a cyanobacterium differentiating only two terminal heterocysts. Additionally, it is shown that the genome of *C. raciborskii* lacks the *pat*L homolog, on which other cyanobacteria with intercalary heterocysts depend. Hence, our study offers baseline information for future research regarding the mechanisms involved in terminal heterocysts differentiation and their assumed highly efficient transfer to the vegetative cells, or detainment of the fixed N_2_ in the trichomes, or a combination of both. This research will also help identify means by which *C. raciborskii* and other terminal heterocyst forming cyanobacteria survive, and as in the case of *C. raciborskii*, even become ecologically successful.

## Materials and Methods

### Strains, culture growth and trichome length determination

Cultures of *C. raciborskii*, and *Anabaena* PCC7120 were obtained from the CSIRO Collection of Living Microalgae and the Pasteur Culture Collection respectively. Cultures were maintained in MLA medium [47], under continuous light or 12 h light 12 h dark (12L/12D) cycles (60 μmol photon m^−2^ s^−1^) and 25°C. For combined nitrogen depletion experiments, cultures were washed with 1 volume of nitrate-free MLA medium (MLA_0_) and resuspended in MLA_0_. To inhibit heterocyst differentiation, cultures were grown, washed and resuspended in MLA_0_ supplemented with 2 mM of NH_4_Cl (MLA_N_). For all experiments, cultures were grown to an OD_750_ of ∼0.20 (measured in a UV-VIS 1240 Mini-spectrometer, Shimadzu).

To determine trichome lengths of diazotrophic *C. raciborskii* cultures (OD_750_ of 0.55–0.65), vegetative cells and heterocysts of 400 trichomes from 4 different cultures were counted by LM.

### Nitrogenase activity and Oxygen concentration assessment

Cultures (10 ml) were transferred to a 17 ml vial, and 1 ml of air was replaced with 1 ml of acetylene. All vials were incubated in agitation for 1 h under the corresponding growth condition. After incubation, 0.5 ml of the gas phase was withdrawn and ethylene content was measured as described in Lundgren *et al*. [48]. Nitrogenase activity rates were normalized to chlorophyll *a* (Chl*a*) or protein content and background ethylene values in the acetylene gas phase were subtracted from control vials lacking cyanobacteria. Oxygen concentration was measured with a pH/oxygen Pocket Meter 340i (WTW) and normalized to protein content. Chl*a* was extracted as described in [49], and proteins were extracted and quantified as described below.

### SDS-PAGE and western-blot analysis

Diazotrophically grown *C. raciborskii* was centrifuged, cells were transferred to an individual lysing matrix tube (Lysing Matrix E, Q Biogene, Noltingham, United Kingdom), and deep frozen in liquid nitrogen. Proteins were extracted and pigments were washed as described previously [50]. Proteins were quantified by RC-DC Protein Assay kit (BioRad, Hercules, CA, USA). For SDS-PAGE, 30 μg of protein was loaded to a 12% (v/v) polyacrylamide separating gel overlaid with a 4% (v/v) stacking gel. Proteins were wet-blotted onto polyvinylidene difluoride membranes (Hybond-P™, Amersham GE Healthcare, Buckinghamshire, UK). Immuno-detection was done as described previously [22], using a chemiluminescent reagent (ECL Plus, GE Healthcare) according to the manufacturer's instructions and visualized using a ChemiDoc XRS system (BioRad). The primary antibodies and the corresponding concentrations utilized were: anti-*Rhodospirillum rubrum* NifH (1∶10000), anti-*Synechocystis* sp. PCC6803 S1 riboprotein (Agrisera, Stockholm, Sweden) (1∶10000), anit-*Anabaena* PCC7120 FtsZ (Agrisera) (1∶1000), or anti-*Synechococcus* PCC 7920 RbcL (Agrisera) (1∶5000).

### Heterocyst and vegetative cell fractionation

To generate the heterocyst and vegetative cell enrichments, we developed a protocol based on the eukaryotic cell partitioning system described by Van Alstine [51] (see Information S1). The efficiency of this cell partition method was revised by counting 100 cells of each fraction by LM, of 4 independent cell partition experiments.

### Nucleic acid extractions and gene expression analysis

Genomic DNA of *C. raciborskii* was extracted according to the CTAB method [52]. For total RNA extraction, diazotrophically grown *C. raciborskii* cultures were collected by vacuum filtration and transferred to lysing matrix tubes with RLT buffer (InviTek, Berlin, Germany). Cells were disrupted and RNA extraction was performed using InviTrap® Spin Cell RNA MiniKit (InviTek) according to the manufacturer's instructions. Samples were DNase treated (turbo DNA-free™, Ambion), and RNA integrity was verified by RNA gel electrophoresis in 1% (w/v) agarose. RNA was reverse transcribed to cDNA using an ImProm-II™ system (Promega, Southampton, United Kingdom), and PCR amplifications were performed using the primers presented in Table S3. PCR amplifications consisted of 35 cycles with annealing at 60°C ×30 s and elongation at 72°C ×1 min. All PCR products were visualized by PAGE 8% (w/v) with TBE, and AgNO_3_ stained [53].

### Immunogold-TEM

Diazotrophically grown *C. raciborskii* and *Anabaena* PCC7120 were sampled 3 h into the light phase of their 12L/12D cycles, concentrated by centrifugation, and embedded in 2% (w/v) agar in phosphate buffer solution (PBS). Agar-embedded trichomes were fixed 30 min in 3% (w/v) paraformaldehyde in PBS, washed 3 times with PBS, dehydrated in an ethanol series (30% to 100% [v/v]), and finally embedded in LR-White™ (London Resin). NifH immunogold labelling was performed as described previously [54], using goat anti-rabbit IgG conjugated to 10-nm gold particles (Sigma-Aldrich) (1∶200 in 10% [w/v] BSA in PBS). Samples were sectioned by ultramicrotome (Sorvall MT5000, Norwalk, CT, USA), and mounted on copper grids for TEM examinations. Control experiments were performed excluding the primary antibody, showing negligible non-specific binding of the secondary antibody to the sections. Statistical differences between the immunogold label in heterocysts and vegetative cells of *C. raciborskii* and *Anabaena* PCC7120 were analyzed by two-way analysis of variance (Statistica 7.0, Statsoft). All samples were observed in an EM 906 transmission electron microscope (Zeiss, Oberkochen, Germany), at 80 kV accelerating voltage.

### Immunofluorescence labelling

Droplets of 30µl of diazotrophic cyanobacteria, grown under 12L/12D cycles, were placed on poly-l-lysine coated microscope glass slides and air dried. Samples were fixed in 70% (v/v) ethanol, at −20°C for 30 min, washed 3×2 min with PBS-T (0.1% [v/v] Tween-20 in PBS), incubated 15 min in blocking solution (3% [w/v] BSA in PBS-T), and for 1.5 h at 4°C with the corresponding primary antibody (1∶100 in blocking solution) in a moisture chamber. Samples were washed with PBS-T, and incubated 45 min at 4°C in a moisture chamber with a secondary goat anti-rabbit IgG antibody conjugated with a fluorescent marker (Alexa-Fluor488, Invitrogen) (diluted to 10 µg ml^−1^ in PBS-T). After washing with PBS-T, samples were covered with a droplet of antifade reagent (ProLong Gold, Invitrogen, Eugene, OR, USA), and visualized using an Axiovert M-200 microscope (Zeiss) equipped with a HRmRev2 camera (Zeiss). The green fluorescent label was visualized by exciting the samples at 488 nm and observed through a 505–530 nm emission filter.

### Phylogenetic analysis of *C. raciborskii patAL* and *hetF*

For the multiple sequence alignments and phylogenetic analyses of PatA, PatL and HetF the corresponding protein sequences in *Anabaena* PCC 7120 (accessions NP_484565, NP_487345 and NP_487586, respectively) were used as queries in a BLASTP [55] search against the non-redundant protein database (nr, v2011-09-28). Homologous sequences with an E-value <1e^−10^and an amino acid (aa) identity ≥30% with a length ≥30% of the query sequence were retrieved (coverage threshold was raised to ≥50% for the PatA search) (Table S2). Multiple sequence alignments were generated using Muscle (v3.8.31, [56]) with default settings. The PatA and HetF alignments were manually edited (Information S1), and sites in alignments with gaps in ≥50% of sequences were removed prior to constructing Maximum Likelihood phylogenetic trees using FastTree v2.1.3 [57] with -slow and -slownni settings. Ratios of non-synonymous and synonymous substitutions for *patA* sequences were calculated in HyPhy (v2.0020110824beta, [58]). Putative transmembrane helices were predicted for all protein sequences using the TMHMM Server v2.0 (http://www.cbs.dtu.dk/services/TMHMM/).

## Supporting Information

Figure S1 **Multiple sequence alignment of PatA homologs in cyanobacteria.** Organism names and protein identifiers are shown in the left margin with the canonical heterocystous sequences shaded in green. The sequences are the same as in Figure 5A. Alignment columns are colored by conserved hydrophobic properties (30% cutoff). The PATAN, linker region and REC domains are indicated from the N- to the C-terminus. The *C. raciborskii* PatA sequence contains both the PATAN and REC domains but has the shortest linker region of the compared sequences.
(JPG)

Information S1 **Supporting Materials and Methods.**
(DOC)

Table S1 **Two-way ANOVA of the immunogold-TEM NifH label in** ***C. raciborskii*** **and** ***Anabaena*** **PCC7120.** df, denotes degrees of freedom; MS, denotes the mean square; * Significant difference.
(DOC)

Table S2 **GeneBank Accession No., gene locus tag and putative protein length of PatAL and HetF cyanobacterial homologs used for phylogenetic analysis.** Information on proteins used for phylogenetic analysis of PatAL and HetF Canonical PatAL and HetF sequences in *Anabaena* sp. PCC 7120 used as queries in BLASTP searches are highlighted green and *Cylindrospermopsis raciborskii* CS-505 and *Raphidiopsis brookii* D9 are highlighted in magenta.
(DOC)

Table S3 **Nucleotide sequences of primers used in this study.** Primer names, sequence and target locus tag in *C. raciborskii* are shown (gene name is given in parentheses).
(DOC)
