# Supplementary figures and images for: Dinitrogen Fixation Is Restricted to the Terminal Heterocysts in the Invasive Cyanobacterium Cylindrospermopsis raciborskii CS-505
Source: PLoS One. 2013 Feb 6;8(2):e51682. doi: 10.1371/journal.pone.0051682 (PMC3566145; doi:10.1371/journal.pone.0051682)

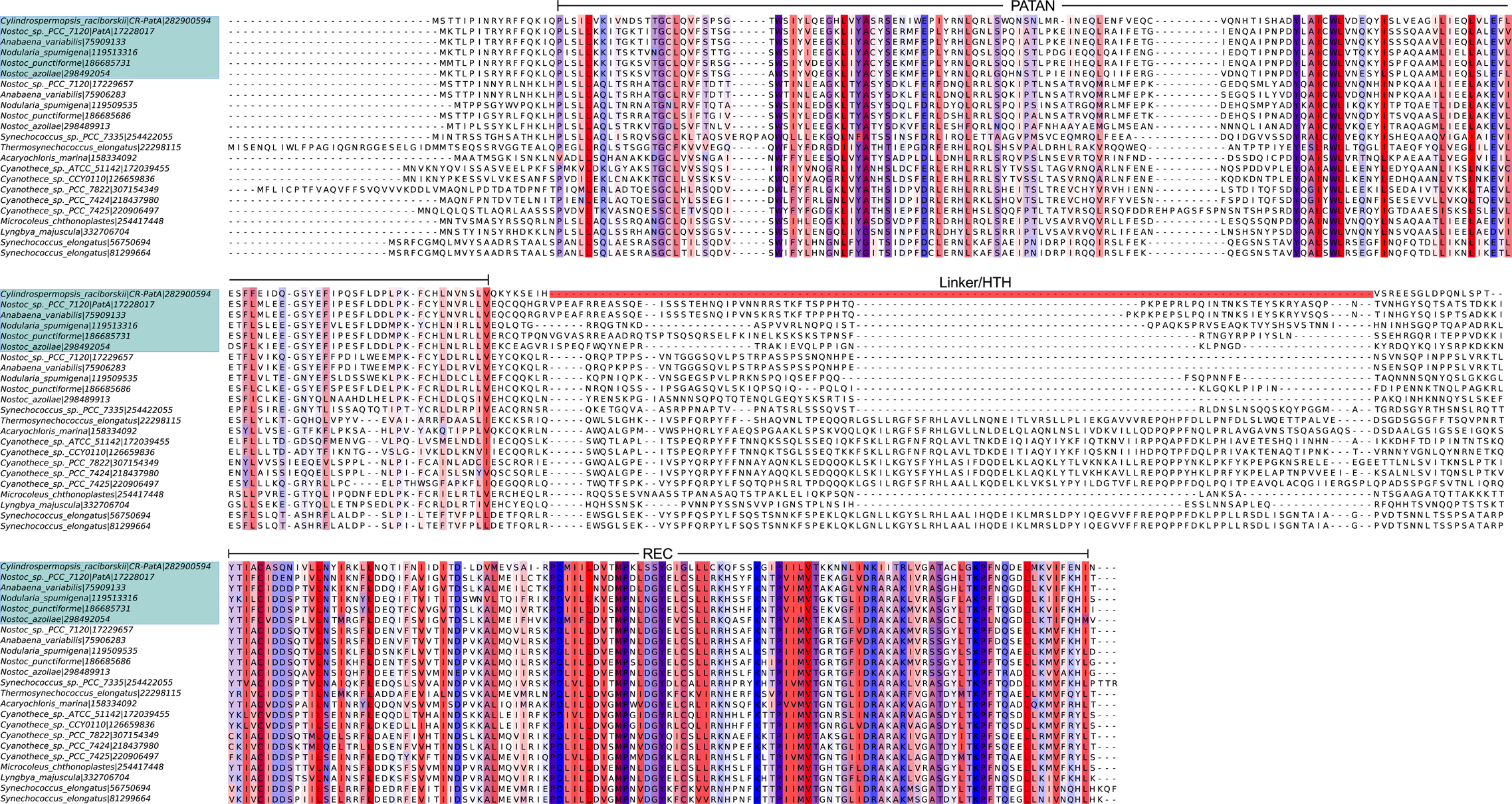

Supplement: Figure S1 — Multiple sequence alignment of PatA homologs in cyanobacteria. Organism names and protein identifiers are shown in the left margin with the canonical heterocystous sequences shaded in green. The sequences are the same as in Figure 5A. Alignment columns are colored by conserved hydrophobic properties (30% cutoff). The PATAN, linker region and REC domains are indicated from the N- to the C-terminus. The C. raciborskii PatA sequence contains both the PATAN and REC domains but has the shortest linker region of the compared sequences. (JPG) [file pone.0051682.s001.jpg]
