# Supplementary material for: Dinitrogen Fixation Is Restricted to the Terminal Heterocysts in the Invasive Cyanobacterium Cylindrospermopsis raciborskii CS-505
Source: PLoS One. 2013 Feb 6;8(2):e51682. doi: 10.1371/journal.pone.0051682 (PMC3566145; doi:10.1371/journal.pone.0051682)
Supplement: Information S1 — Supporting Materials and Methods. (DOC) [file pone.0051682.s002.doc]

**Supporting Materials and Methods**

Heterocyst and vegetative cell portioning system

The partitioning systems were prepared with fresh stock solutions of: 20 % (w/v) Dextran 500 KDa; 30 % (w/v) polyethylene glycol (PEG) of 8 KDa; potassium phosphate buffer (218 mM K_2_HPO_4_ and 69 mM KH_2_PO_4_, pH 7.2); and, 600 mM KCl. An aqueous polymer mixture of 33 g was prepared, resulting in a two-phase aqueous polymer system of 7 % (w/v) Dextran 500 KDa, 7 % (w/v) PEG 8 KDa, 150 mM KCl, 7.3 mM K_2_HPO_4_ and 2.3 mM KH_2_PO_4_. For all enrichments 1.5 l of *C. raciborskii*, grown diazotrophically under continuous light, were concentrated by vacuum filtration and sonicated 5 × 5 s at 80 % amplitude (70 W HF-output, Sonopulse Bandelin, UW 2070). The disrupted filaments were concentrated by centrifugation, the supernatant was discarded and the pellet was resuspended in the upper phase of the partitioning system. Each partitioning system was mixed by gently inverting the tubes 10 times,and left to separate until a green upper phase and a brownish-yellow lower phase were clearly distinguishable (generally after 20 min). Upper and lower phases were collected separately and repartitioned in the complementary phase of a new 7 % polymer system. The new two-phase systems were mixed by gently inverting the tubes 10 times and left to separate for 30 min. The upper and lower phases were collected and centrifuged at 8000 ***g*** for 15 min. The supernatant was discarded and the enriched heterocyst (lower phase) and vegetative cells (upper phase) were resuspended in MLA_0_. When RNA or protein extraction were performed, the cell enrichments were transferred to an individual lysing matrix tube, frozen in liquid nitrogen and left at -80 ºC for further use.

Phylogenetic analysis of *C. raciborskii pat*AL and *het*F*.*

The PatA and HetF alignments were manually edited in Jalview [59] to correct the position of gaps. For PatA, manual editing was aided by secondary structure predictions inferred from the JNet function [60] implemented in Jalview with the *Anabaena*  PCC 7120 PatA sequence as query.

Further sequence editing was done to three of the HetF sequences, including the query sequence from A*nabaena* PCC7120. The A*nabaena* PCC7120 HetF protein (hereafter N7120-HetF) is annotated as a 779 aa long sequence encoded by the gene *alr3546*. However, our analysis revealed that, in addition to the putative N-terminal CHAT (PF12770) domain, N7120-HetF and most of the 42 homologues retrieved contained an N-terminal stretch of approximately 50 conserved aa which were missing from the N7120-HetF annotation. Since the upstream genomic region of N7120-*hetF* contained this conserved region in the same uninterrupted reading frame as the annotated *hetF* sequence, these missing aa were included in further analysis. The analysis showed that the HetF homolog of the filamentous but non-heterocystous *Raphidiopsis brookii* D9 was encoded by the neighboring genes *CRD_00315* and *CRD_00314* due to a putative frameshift, or sequencing error, in the *R. brookii* *hetF* nucleotide sequence. Both gene products were concatenated to an 801 aa long protein which was used in further analyses. Additionally, a short *hetF* homolog in *Trichodesmium erythraeum* was excluded from the subsequent analysis as it showed significant similarity to only the C-terminal parts of the other sequences and appeared to be a duplication of a longer and neighouring *hetF* homolog in this genome.

**References**

1. Waterhouse AM, Procter JB, Martin DMA, Clamp M, Barton GJ. (2009) Jalview Version 2--a multiple sequence alignment editor and analysis workbench. Bioinformatics 25: 1189-1191.
2. Cole C, Barber JD, Barton GJ. (2008) The Jpred 3 secondary structure prediction server. Nucleic Acids Res 36: W197-201.
