# Supplementary material for: Dinitrogen Fixation Is Restricted to the Terminal Heterocysts in the Invasive Cyanobacterium Cylindrospermopsis raciborskii CS-505
Source: PLoS One. 2013 Feb 6;8(2):e51682. doi: 10.1371/journal.pone.0051682 (PMC3566145; doi:10.1371/journal.pone.0051682)
Supplement: Supplementary file 3 [file pone.0051682.s003.doc]

| Source of Variance | df | MS | df | MS |  |  |
| --- | --- | --- | --- | --- | --- | --- |
|  | Effect | Effect | Error | Error | F-value | p-value |
| Different Cell Type | 1 | 649152 | 88 | 3864,1352 | 167,9941 | 0,0001* |
| Different Cyanobacteria | 1 | 1520,3913 | 88 | 3864,1352 | 0,3934 | 0,5321 |
| Same cell type but of different Cyanobacteria | 1 | 84,1739 | 88 | 3864,1352 | 0,0217 | 0,8830 |
