# Supplementary material for: Dinitrogen Fixation Is Restricted to the Terminal Heterocysts in the Invasive Cyanobacterium Cylindrospermopsis raciborskii CS-505
Source: PLoS One. 2013 Feb 6;8(2):e51682. doi: 10.1371/journal.pone.0051682 (PMC3566145; doi:10.1371/journal.pone.0051682)
Supplement: Supplementary file 4 [file pone.0051682.s004.doc]

| PatA |  |  |  |  |  |
| --- | --- | --- | --- | --- | --- |
| Organism | Organism abbreviation | Locus tag | Protein accession | Protein GI number | Length |
|  |  |  |  |  |  |
| 'Nostoc azollae' 0708 | NoAz | Aazo_0413 | YP_003720090.1 | 298489913 | 347 |
| 'Nostoc azollae' 0708 | NoAz | Aazo_3491 | YP_003722231.1 | 298492054 | 345 |
| Acaryochloris marina MBIC11017 | Acam | AM1_0908 | YP_001515264.1 | 158334092 | 409 |
| Anabaena variabilis ATCC 29413 | Anav | Ava_0058 | YP_320579.1 | 75906283 | 347 |
| Anabaena variabilis ATCC 29413 | Anav | Ava_2923 | YP_323429.1 | 75909133 | 381 |
| Cyanothece sp. ATCC 51142 | Cya51142 | cce_4542 | YP_001805956.1 | 172039455 | 416 |
| Cyanothece sp. CCY0110 | Cya0110 | CY0110_24426 | ZP_01730962.1 | 126659836 | 416 |
| Cyanothece sp. PCC 7424 | Cya7424 | PCC7424_0987 | YP_002376309.1 | 218437980 | 414 |
| Cyanothece sp. PCC 7425 | Cya7425 | Cyan7425_1061 | YP_002481808.1 | 220906497 | 427 |
| Cyanothece sp. PCC 7822 | Cya7822 | Cyan7822_4548 | YP_003889733.1 | 307154349 | 439 |
| Cylindrospermopsis raciborskii CS-505 | CS505 | CRC_01956 | ZP_06308536.1 | 282900594 | 308 |
| Lyngbya majuscula 3L | Lynmaj | LYNGBM3L_23230 | ZP_08426765.1 | 332706704 | 356 |
| Microcoleus chthonoplastes PCC 7420 | Micc | MC7420_1558 | ZP_05031189.1 | 254417448 | 355 |
| Nodularia spumigena CCY9414 | Nods | N9414_13295 | ZP_01632354.1 | 119513316 | 358 |
| Nodularia spumigena CCY9414 | Nods | N9414_08215 | ZP_01628682.1 | 119509535 | 351 |
| Nostoc punctiforme PCC 73102 | Nosp | Npun_F5682 | YP_001868927.1 | 186685731 | 367 |
| Nostoc punctiforme PCC 73102 | Nosp | Npun_F5636 | YP_001868882.1 | 186685686 | 355 |
| Anabaena sp. PCC 7120 | Noss | all0521 | NP_484565.1 | 17228017 | 379 |
| Anabaena sp. PCC 7120 | Noss | all2165 | NP_486205.1 | 17229657 | 347 |
| Synechococcus elongatus PCC 6301 | Syne6301 | syc0685_c | YP_171395.1 | 56750694 | 419 |
| Synechococcus elongatus PCC 7942 | Syne7942 | Synpcc7942_0855 | YP_399872.1 | 81299664 | 417 |
| Synechococcus sp. PCC 7335 | Syne7335 | S7335_2205 | ZP_05035773.1 | 254422055 | 369 |
| Thermosynechococcus elongatus BP-1 | Thee | tll0572 | NP_681362.1 | 22298115 | 435 |
|  |  |  |  |  |  |
|  |  |  |  |  |  |
|  |  |  |  |  |  |
| PatL |  |  |  |  |  |
| Organism | Organism abbreviation | Locus tag | Protein accession | Protein GI number | Length |
|  |  |  |  |  |  |
| 'Nostoc azollae' 0708 | NoAz | Aazo_3834 | YP_003722478.1 | 298492301 | 264 |
| 'Nostoc azollae' 0708 | NoAz | Aazo_0677 | YP_003720268.1 | 298490091 | 214 |
| 'Nostoc azollae' 0708 | NoAz | Aazo_1101 | YP_003720574.1 | 298490397 | 240 |
| 'Nostoc azollae' 0708 | NoAz | Aazo_1100 | YP_003720573.1 | 298490396 | 493 |
| 'Nostoc azollae' 0708 | NoAz | Aazo_1544 | YP_003720858.1 | 298490681 | 706 |
| 'Nostoc azollae' 0708 | NoAz | Aazo_0129 | YP_003719895.1 | 298489718 | 182 |
| Acaryochloris marina MBIC11017 | Acam | AM1_B0195 | YP_001521229.1 | 158340059 | 483 |
| Anabaena variabilis ATCC 29413 | Anav | Ava_3427 | YP_323929.1 | 75909633 | 365 |
| Anabaena variabilis ATCC 29413 | Anav | Ava_1585 | YP_322103.1 | 75907807 | 237 |
| Anabaena variabilis ATCC 29413 | Anav | Ava_0605 | YP_321124.1 | 75906828 | 727 |
| Anabaena variabilis ATCC 29413 | Anav | Ava_4963 | YP_325455.1 | 75911159 | 489 |
| Anabaena variabilis ATCC 29413 | Anav | Ava_4964 | YP_325456.1 | 75911160 | 250 |
| Anabaena variabilis ATCC 29413 | Anav | Ava_4189 | YP_324683.1 | 75910387 | 222 |
| Anabaena variabilis ATCC 29413 | Anav | Ava_1254 | YP_321773.1 | 75907477 | 213 |
| Anabaena variabilis ATCC 29413 | Anav | Ava_0290 | YP_320811.1 | 75906515 | 182 |
| Anabaena variabilis ATCC 29413 | Anav | Ava_0211 | YP_320732.1 | 75906436 | 219 |
| Anabaena variabilis ATCC 29413 | Anav | Ava_4398 | YP_324891.1 | 75910595 | 521 |
| Anabaena variabilis ATCC 29413 | Anav | Ava_4910 | YP_325402.1 | 75911106 | 268 |
| Anabaena variabilis ATCC 29413 | Anav | Ava_4082 | YP_324581.1 | 75910285 | 189 |
| Anabaena variabilis ATCC 29413 | Anav | Ava_4850 | YP_325342.1 | 75911046 | 576 |
| Arthrospira maxima CS-328 | Amax | AmaxDRAFT_3273 | ZP_03274449.1 | 209525915 | 760 |
| Arthrospira platensis str. Paraca | Artp | AplaP_010100001715 | ZP_06380380.1 | 284050170 | 741 |
| Crocosphaera watsonii WH 8501 | Crow | CwatDRAFT_0827 | ZP_00518858.1 | 67925523 | 273 |
| cyanobacterium UCYN-A | Ucyn | UCYN_11960 | YP_003422245.1 | 284929723 | 243 |
| Cyanobium sp. PCC 7001 | Cya7001 | CPCC7001_1293 | ZP_05045105.1 | 254431402 | 221 |
| Cyanothece sp. ATCC 51142 | Cya51152 | cce_3979 | YP_001805393.1 | 172038892 | 335 |
| Cyanothece sp. CCY0110 | Cya0110 | CY0110_23806 | ZP_01727932.1 | 126656718 | 330 |
| Cyanothece sp. PCC 7424 | Cya7424 | PCC7424_1025 | YP_002376347.1 | 218438018 | 333 |
| Cyanothece sp. PCC 7425 | Cya7425 | Cyan7425_5254 | YP_002485907.1 | 220910596 | 449 |
| Cyanothece sp. PCC 7822 | Cya7822 | Cyan7822_2726 | YP_003887968.1 | 307152584 | 333 |
| Cyanothece sp. PCC 8801 | Cya8801 | PCC8801_3419 | YP_002373543.1 | 218248172 | 336 |
| Cyanothece sp. PCC 8802 | Cya8802 | Cyan8802_0392 | YP_003136187.1 | 257058299 | 291 |
| Cylindrospermopsis raciborskii CS-505 | CS505 | CRC_00125 | ZP_06306820.1 | 282898833 | 189 |
| Cylindrospermopsis raciborskii CS-505 | CS505 | CRC_00361 | ZP_06307031.1 | 282899050 | 268 |
| Cylindrospermopsis raciborskii CS-505 | CS505 | CRC_03400 | ZP_06309861.1 | 282901962 | 275 |
| Cylindrospermopsis raciborskii CS-505 | CS505 | CRC_00304 | ZP_06307006.1 | 282899024 | 224 |
| Cylindrospermopsis raciborskii CS-505 | CS505 | CRC_00692 | ZP_06306699.1 | 282898711 | 682 |
| Fremyella diplosiphon | Fremy |  | AAQ63050.1 | 34223765 | 238 |
| Gloeobacter violaceus PCC 7421 | Glov | gll2892 | NP_925838.1 | 37522461 | 457 |
| Leptolyngbya valderiana BDU 20041 | Lepv | LVAL_00031 | ZP_01811409.1 | 148972698 | 170 |
| Lyngbya majuscula 3L | Lymaj | LYNGBM3L_00660 | ZP_08425038.1 | 332704952 | 544 |
| Lyngbya sp. PCC 8106 | Lyn8106 | L8106_16994 | ZP_01620430.1 | 119486371 | 772 |
| Microcoleus chthonoplastes PCC 7420 | Micc | MC7420_162 | ZP_05031376.1 | 254417642 | 436 |
| Microcoleus vaginatus FGP-2 | Micv |  | ZP_08491199.1 | 334117107 | 520 |
| Microcystis aeruginosa PCC 7806 | Mica | IPF_2345 | CAO89998.1 | 159028827 | 481 |
| Nodularia spumigena CCY9414 | Nods | N9414_23018 | ZP_01630685.1 | 119511578 | 226 |
| Nodularia spumigena CCY9414 | Nods | N9414_05989 | ZP_01630781.1 | 119511676 | 498 |
| Nodularia spumigena CCY9414 | Nods | N9414_05994 | ZP_01630782.1 | 119511677 | 249 |
| Nodularia spumigena CCY9414 | Nods | N9414_09151 | ZP_01631348.1 | 119512260 | 715 |
| Nodularia spumigena CCY9414 | Nods | N9414_17453 | ZP_01628639.1 | 119509491 | 284 |
| Nodularia spumigena CCY9414 | Nods | N9414_10852 | ZP_01631617.1 | 119512539 | 185 |
| Nodularia spumigena CCY9414 | Nods | N9414_09756 | ZP_01632333.1 | 119513293 | 373 |
| Nodularia spumigena CCY9414 | Nods | N9414_13235 | ZP_01631839.1 | 119512769 | 268 |
| Nodularia spumigena CCY9414 | Nods | N9414_10173 | ZP_01630005.1 | 119510881 | 211 |
| Nostoc punctiforme | Nosp |  | 2J8K_A | 122920845 | 201 |
| Nostoc punctiforme PCC 73102 | Nosp | Npun_R2559 | YP_001866056.1 | 186682860 | 589 |
| Nostoc punctiforme PCC 73102 | Nosp | Npun_F0732 | YP_001864427.1 | 186681231 | 282 |
| Nostoc punctiforme PCC 73102 | Nosp | Npun_F1220 | YP_001864887.1 | 186681691 | 218 |
| Nostoc punctiforme PCC 73102 | Nosp | Npun_R3900 | YP_001867226.1 | 186684030 | 212 |
| Nostoc punctiforme PCC 73102 | Nosp | Npun_F4767 | YP_001868061.1 | 186684865 | 491 |
| Nostoc punctiforme PCC 73102 | Nosp | Npun_R1555 | YP_001865187.1 | 186681991 | 268 |
| Nostoc punctiforme PCC 73102 | Nosp | Npun_R5810 | YP_001869051.1 | 186685855 | 431 |
| Nostoc punctiforme PCC 73102 | Nosp | Npun_F4691 | YP_001867990.1 | 186684794 | 544 |
| Nostoc punctiforme PCC 73102 | Nosp | Npun_F0979 | YP_001864653.1 | 186681457 | 539 |
| Nostoc punctiforme PCC 73102 | Nosp | Npun_R4204 | YP_001867522.1 | 186684326 | 413 |
| Nostoc punctiforme PCC 73102 | Nosp | Npun_F3247 | YP_001866633.1 | 186683437 | 176 |
| Nostoc punctiforme PCC 73102 | Nosp | Npun_F4766 | YP_001868060.1 | 186684864 | 247 |
| Nostoc punctiforme PCC 73102 | Nosp | Npun_F6541 | YP_001869744.1 | 186686548 | 330 |
| Nostoc punctiforme PCC 73102 | Nosp | Npun_F0318 | YP_001864046.1 | 186680850 | 717 |
| Nostoc punctiforme PCC 73102 | Nosp | Npun_R5143 | YP_001868418.1 | 186685222 | 214 |
| Anabaena sp. PCC 7120 | N7120 | alr1746 | NP_485786.1 | 17229238 | 182 |
| Anabaena sp. PCC 7120 | N7120 |  | 3DU1_X | 212374999 | 257 |
| Anabaena sp. PCC 7120 | N7120 | all3114 | NP_487154.1 | 17230606 | 576 |
| Anabaena sp. PCC 7120 | N7120 | all3869 | NP_487909.1 | 17231361 | 376 |
| Anabaena sp. PCC 7120 | N7120 | all4303 | NP_488343.1 | 17231795 | 213 |
| Anabaena sp. PCC 7120 | N7120 | all3306 | NP_487346.1 | 17230798 | 252 |
| Anabaena sp. PCC 7120 | N7120 | all3305 | NP_487345.1 | 17230797 | 496 |
| Anabaena sp. PCC 7120 | N7120 | all3740 | NP_487780.1 | 17231232 | 237 |
| Anabaena sp. PCC 7120 | N7120 | alr1579 | NP_485619.1 | 17229071 | 222 |
| Anabaena sp. PCC 7120 | N7120 | all0813 | NP_484856.1 | 17228308 | 727 |
| Anabaena sp. PCC 7120 | N7120 | alr1142 | NP_485185.1 | 17228637 | 521 |
| Anabaena sp. PCC 7120 | N7120 | all2395 | NP_486435.1 | 17229887 | 222 |
| Anabaena sp. PCC 7120 | N7120 | all3256 | NP_487296.1 | 17230748 | 268 |
| Oscillatoria sp. PCC 6506 | Osc6506 | OSCI_3310010 | ZP_07111911.1 | 300867251 | 520 |
| Raphidiopsis brookii D9 | Rapb | CRD_01938 | ZP_06305021.1 | 282897019 | 224 |
| Raphidiopsis brookii D9 | Rapb | CRD_01636 | ZP_06304938.1 | 282896932 | 689 |
| Raphidiopsis brookii D9 | Rapb | CRD_02410 | ZP_06305842.1 | 282897847 | 222 |
| Raphidiopsis brookii D9 | Rapb | CRD_03055 | ZP_06306506.1 | 282898516 | 268 |
| Raphidiopsis brookii D9 | Rapb | CRD_01568 | ZP_06304870.1 | 282896864 | 189 |
| Synechococcus sp. CB0101 | SynCB101 | SCB01_010100009469 | ZP_07973885.1 | 318041929 | 208 |
| Synechococcus sp. CB0205 | SynCB205 | SCB02_010100004258 | ZP_07970121.1 | 317968731 | 223 |
| Synechococcus sp. JA-2-3B'a(2-13) | YellB | CYB_1346 | YP_477582.1 | 86608820 | 328 |
| Synechococcus sp. JA-3-3Ab | YellA | CYA_0793 | YP_474262.1 | 86605499 | 330 |
| Synechococcus sp. PCC 7002 | Syn7002 | SYNPCC7002_A1220 | YP_001734474.1 | 170077836 | 330 |
| Synechococcus sp. PCC 7335 | Syn7335 | S7335_2507 | ZP_05036075.1 | 254422357 | 346 |
| Synechococcus sp. RS9917 | Syn9917 | RS9917_12965 | ZP_01080374.1 | 87124525 | 221 |
| Synechocystis sp. PCC 6803 | Syne6803 | slr1819 | NP_441076.1 | 16330348 | 331 |
| Thermosynechococcus elongatus BP-1 | Thee | tll0709 | NP_681498.1 | 22298251 | 449 |
| Trichodesmium erythraeum IMS101 | Trie | Tery_2700 | YP_722362.1 | 113476301 | 517 |
|  |  |  |  |  |  |
|  |  |  |  |  |  |
|  |  |  |  |  |  |
| HetF |  |  |  |  |  |
| Organism | Organism abbreviation | Locus tag | Protein accession | Protein GI number | Length |
|  |  |  |  |  |  |
| 'Nostoc azollae' 0708 | NoAz | Aazo_0033 | YP_003719813.1 | 298489636 | 823 |
| 'Nostoc azollae' 0708 | NoAz | Aazo_2711 | YP_003721703.1 | 298491526 | 544 |
| Anabaena variabilis ATCC 29413 | Anav | Ava_0305 | YP_320826.1 | 75906530 | 525 |
| Anabaena variabilis ATCC 29413 | Anav | Ava_3525 | YP_324027.1 | 75909731 | 835 |
| Arthrospira maxima CS-328 | Amax | AmaxDRAFT_2530 | ZP_03273710.1 | 209525167 | 814 |
| Arthrospira maxima CS-328 | Amax | AmaxDRAFT_2831 | ZP_03274007.1 | 209525467 | 565 |
| Arthrospira platensis NIES-39 | Artp | NIES39_J02400 | BAI91288.1 | 291569016 | 826 |
| Arthrospira platensis NIES-39 | Artp | NIES39_A02030 | BAI88042.1 | 291565770 | 562 |
| Arthrospira platensis str. Paraca | Artp | AplaP_010100021916 | ZP_06384320.1 | 284054110 | 458 |
| Arthrospira platensis str. Paraca | Artp | AplaP_010100006675 | ZP_06381351.1 | 284051141 | 827 |
| Cyanothece sp. PCC 7424 | Cya7424 | PCC7424_3255 | YP_002378523.1 | 218440194 | 569 |
| Cyanothece sp. PCC 7822 | Cya7822 | Cyan7822_3439 | YP_003888663.1 | 307153279 | 598 |
| Cyanothece sp. PCC 8801 | Cya8801 | PCC8801_3407 | YP_002373531.1 | 218248160 | 562 |
| Cyanothece sp. PCC 8802 | Cya8802 | Cyan8802_2709 | YP_003138404.1 | 257060516 | 562 |
| Cylindrospermopsis raciborskii CS-505 | CS505 | CRC_00037 | ZP_06306733.1 | 282898746 | 512 |
| Cylindrospermopsis raciborskii CS-505 | CS505 | CRC_02207 | ZP_06308282.1 | 282900331 | 787 |
| Lyngbya majuscula 3L | Lymaj | LYNGBM3L_33580 | ZP_08428998.1 | 332709028 | 842 |
| Lyngbya majuscula 3L | Lymaj | LYNGBM3L_19530 | ZP_08428365.1 | 332708388 | 544 |
| Lyngbya sp. PCC 8106 | Lyn8106 | L8106_04751 | ZP_01618747.1 | 119483333 | 561 |
| Lyngbya sp. PCC 8106 | Lyn8106 | L8106_20675 | ZP_01622657.1 | 119490033 | 817 |
| Microcoleus chthonoplastes PCC 7420 | Micc | MC7420_6879 | ZP_05030097.1 | 254416344 | 543 |
| Microcoleus chthonoplastes PCC 7420 | Micc | MC7420_321 | ZP_05024621.1 | 254410843 | 850 |
| Microcoleus vaginatus FGP-2 | Micv | MicvaDRAFT_0677 | ZP_08494059.1 | 334119976 | 566 |
| Microcoleus vaginatus FGP-2 | Micv | MicvaDRAFT_4878 | ZP_08494239.1 | 334120157 | 612 |
| Microcystis aeruginosa NIES-843 | Mica | MAE_25310 | YP_001657545.1 | 166365272 | 550 |
| Microcystis aeruginosa PCC 7806 | Mica | IPF_4097 | CAO90746.1 | 159029370 | 551 |
| Nodularia spumigena CCY9414 | Nods | N9414_06174 | ZP_01630733.1 | 119511627 | 845 |
| Nodularia spumigena CCY9414 | Nods | N9414_05339 | ZP_01629862.1 | 119510735 | 521 |
| Nostoc punctiforme PCC 73102 | Nosp | Npun_F1520 | YP_001865153.1 | 186681957 | 526 |
| Nostoc punctiforme PCC 73102 | Nosp | Npun_F3553 | YP_001866897.1 | 186683701 | 834 |
| Anabaena sp. PCC 7120 | N7120 | all1730 | NP_485770.1 | 17229222 | 525 |
| Anabaena sp. PCC 7120 | N7120 | alr3546 | NP_487586.1 | 17231038 | 779 |
| Oscillatoria sp. PCC 6506 | Osc6506 | OSCI_3520035 | ZP_07112637.1 | 300867998 | 563 |
| Oscillatoria sp. PCC 6506 | Osc6506 | OSCI_1900018 | ZP_07110290.1 | 300865499 | 847 |
| Raphidiopsis brookii D9 | Rapb | CRD_00314/CRD_00315 | CB237991.1 | 28289569[01] | 794 |
| Raphidiopsis brookii D9 | Rapb | CRD_02582 | ZP_06306011.1 | 282898016 | 512 |
| Synechococcus sp. PCC 7335 | Syn7335 | S7335_3754 | ZP_05037316.1 | 254423598 | 799 |
| Synechococcus sp. PCC 7335 | Syn7335 | S7335_2970 | ZP_05036536.1 | 254422818 | 540 |
| Trichodesmium erythraeum IMS101 | Trie | Tery_4219 | YP_723693.1 | 113477632 | 806 |
| Trichodesmium erythraeum IMS101 | Trie | Tery_1337 | YP_721126.1 | 113475065 | 559 |
