# Supplementary material for: Dinitrogen Fixation Is Restricted to the Terminal Heterocysts in the Invasive Cyanobacterium Cylindrospermopsis raciborskii CS-505
Source: PLoS One. 2013 Feb 6;8(2):e51682. doi: 10.1371/journal.pone.0051682 (PMC3566145; doi:10.1371/journal.pone.0051682)
Supplement: Supplementary file 5 [file pone.0051682.s005.doc]

| Primer | Sequence (5’-3’) | Gene Target |
| --- | --- | --- |
| CS505nifHF | GGCTGCTGAGAAAGGTGCTGT | CRC_02887 (nifH) |
| CS505nifHR | ACCACGACCAGCACAACCT | CRC_02887 (nifH) |
| CS505devAF | TTAAGTTATGGTGGAGGAGTCG | CRC_00436 (devA) |
| CS505devAR | GTTACCCATGATAATCGCATCC | CRC_00436 (devA) |
| CS505hglDF | AGCCATTATTGGATTATCATGTCTC | CRC_02049 (hglD) |
| CS505hglDR | TGGCAGTTGCATCCTTTTCTTG | CRC_02049 (hglD) |
| RBD916SF | CCGCCAGCTTTTCAGAATAC | 16S rRNA gene |
| RBD916SR | CTCAAGCGGCTCTAATTTGC | 16S rRNA gene |
| CS505ftsZF | GGTAATCCCTCCATTGGTCAA | CRC_03281 (ftsZ) |
| CS505ftsZR | AGCACCCGTACCTGTACCAC | CRC_03281 (ftsZ) |
